# Intraperitoneal Paclitaxel-Induced Eosinophil Recruitment as a Potential Mediator of Tumor Response in Peritoneal Metastases from Gastric Cancer

**DOI:** 10.1245/s10434-025-19075-x

**Published:** 2026-01-29

**Authors:** Misaki Matsumiya, Hirofumi Sonoda, Hiroharu Yamashita, Kentaro Kurashina, Kazuya Takahashi, Hideyuki Ohzawa, Hideyo Miyato, Shiro Matsumoto, Shin Saito, Yoshinori Hosoya, Naohiro Sata, Joji Kitayama, Hironori Yamaguchi

**Affiliations:** 1 Department of Gastrointestinal Surgery, Jichi Medical University, Shimotsuke, Japan; 2 Department of Clinical Oncology, Jichi Medical University, Shimotsuke, Japan; 3 Department of Surgical Oncology, Japan Institute of Health Security, Shinjuku, Tokyo, Japan

**Keywords:** Peritoneal metastasis, Intraperitoneal chemotherapy, Gastric cancer, Paclitaxel, Eosinophil, Apoptosis, Peritoneal cytology

## Abstract

**Background and Purpose:**

The peritoneal cavity constitutes a unique immune microenvironment that critically influences the pathobiology of peritoneal metastasis (PM). This study aimed to clarify the mechanisms by which local immune alterations affect the efficacy of intraperitoneal (IP) chemotherapy for PM from gastric cancer (GC).

**Method:**

Peritoneal lavage or ascitic fluid was obtained from 42 patients with GC and PM treated with IP paclitaxel (PTX) in combination with systemic oxaliplatin and oral S-1. Serial samples from 31 patients were analyzed after 1–3 cycles of chemotherapy. Immune cell subsets were evaluated using multicolor flow cytometry with monoclonal antibodies, and the functional properties of peritoneal eosinophils were assessed using gene expression profiling and cytotoxicity assays.

**Results:**

IP chemotherapy was associated with decreased CD4(+) T cells and increased CD11b(+) myeloid cells. Notably, many patients, particularly those with negative cytology (CY0), exhibited striking recruitment of CD66b(+) CD16(–) CD193(+) Siglec-F(+) eosinophils into the peritoneal cavity. Eosinophil expansion was correlated with improved clinical outcomes. Post-treatment eosinophils displayed an activated, partially degranulated phenotype with elevated CD11b and CD63 expression and distinct messenger RNA signatures compared with circulating eosinophils. Peritoneal eosinophils demonstrated the ability to induce apoptosis in GC cells.

**Conclusion:**

IP PTX promotes the recruitment and activation of eosinophils with potent antitumor activity in the peritoneal cavity. Early post-treatment abdominal eosinophilia is a robust prognostic biomarker and may represent a promising therapeutic target to enhance the efficacy of IP chemotherapy in patients with PM from GC.

**Supplementary Information:**

The online version contains supplementary material available at 10.1245/s10434-025-19075-x.

Gastric cancer (GC) is a significant contributor to cancer-related morbidity and mortality, ranking as the fifth most frequently diagnosed cancer and fourth leading cause of cancer-related deaths worldwide.^1^ Peritoneal metastases (PM) are a common recurrence in patients with GC, particularly in the scirrhous type involving serosal exposure.^2,3^ Currently, patients with PM are generally treated with systemic chemotherapy, similar to those with metastases at other sites. However, PM often demonstrates resistance to systemic chemotherapy, likely due to the "peritoneal–plasma barrier," which impedes effective drug delivery from the systemic circulation to peritoneal lesions.^4^ Paclitaxel (PTX), characterized by its hydrophobic properties and high molecular weight, exhibits prolonged retention in the peritoneal cavity when administered intraperitoneally (IP). This allows for sustained high concentrations within the peritoneal cavity, facilitating direct infiltration into peritoneal nodules, causing notable antitumor activity against peritoneal deposits.^5,6^ Recent studies have demonstrated that the combination of IP administration of PTX with systemic chemotherapy produces remarkable responses in peritoneal lesions and improves outcomes in patients with PM from GC.^7,8^

Over the past decade, cancer immunotherapy with immune checkpoint inhibitors has emerged as a potent and effective therapeutic strategy for treating various malignancies, highlighting the critical role of host immunity in tumor response.^9,10^ Consequently, the tumor immune microenvironment has gained recognition as a pivotal factor that influences the efficacy of chemotherapy.^11,12^ The peritoneal cavity, the largest anatomical space in the human body, possesses immunological characteristics that are distinct from systemic immunity. Lined by a single layer of mesothelial cells, the peritoneum contains a small volume of fluid enriched with resident macrophages, B-1 cells, predominantly CD8(+) over CD4(+) T cells, and a variety of soluble factors.^13–15^ Although these immune cells are thought to play a protective role in suppressing peritoneal tumor growth, it has been hypothesized that peritoneal immunity undergoes significant alterations during IP chemotherapy with PTX. However, the relationship between peritoneal immunity and the tumor response to IP chemotherapy remains poorly characterized, necessitating further investigation.

In this study, we employed flow cytometry to analyze the changes in immune cell composition in the peritoneal fluid of patients with PM from GC who underwent combination chemotherapy with IP PTX. Interestingly, a remarkable increase in eosinophil counts within the abdominal cavity was observed following treatment in patients who exhibited excellent therapeutic responses. Subsequently, we investigated the phenotype, messenger RNA expression and functional roles of these eosinophils and evaluated their impact on patient outcomes.

## Materials and Methods

### Patient and Samples

A total of 42 patients with PM from GC were treated with S1+oxaliplatin and IP PTX using a peritoneal access port in the Department of Gastrointestinal Surgery, Jichi Medical University, Japan, from June 2020 to April 2025. PTX was diluted in 1 L of normal saline and IP administered through the port at 40 mg/m^2^ on days 1 and 8 based on the results of a previous study. Oxaliplatin was intravenously (IV) administered at 100 mg/m^2^ on day 1 and S-1 was administered at 80 mg/m^2^ for 14 consecutive days, followed by 7 days of rest.^16^

Single-cell suspensions were obtained from ascitic fluid or peritoneal lavage samples collected prior to the initiation of IP chemotherapy. In patients presenting with substantial ascites, 10–20 mL of fluid was aspirated via paracentesis. In patients without clinically detectable ascites, the peritoneal cavity was irrigated with 500 mL of sterile normal saline during diagnostic laparoscopy, and 250 mL of recovered fluid was collected prior to any surgical manipulation. Peritoneal fluid samples were also collected using the same procedure through indwelling peritoneal access ports following one course of IP chemotherapy in 27 patients, and after two or three courses in an additional four patients. This study was conducted in accordance with the Declaration of Helsinki and approved by the Ethics Committee of Jichi Medical University (RIN21-HEN005)

### Monoclonal Antibodies

Fluorescein isothiocyanate (FITC)-conjugated monoclonal antibodies (mAbs) to CD45, and CD4; phycoerythrin (PE)-conjugated mAbs to CD56, CD16, and CD326 (EpCAM); allophycocyanin (APC)-conjugated mAbs to CD8 and CD14; Siglec-8; BV605-conjugated mAbs to CD3 and CD11b; PE-conjugated mAbs to CCR-3 (CD193); and BV711-conjugated mAbs to CD163, CD19, and CD63 were purchased from BioLegend (San Diego, CA, USA). FITC-conjugated mAbs to CD66b were purchased from Bio-Rad (Hercules, CA, USA). Fc-blocker and DAPI were purchased from Thermo Fisher Scientific (Waltham, MA, USA), and FVS780 from Becton-Dickinson (San Jose, CA, USA).

### Flow Cytometry

After centrifugation of the ascites or peritoneal lavage fluid at 1500 rpm for 10 min, the pellets were resuspended and washed with phosphate buffered saline (PBS)+0.02% ethylenediaminetetraacetic acid (EDTA). During this procedure, most of the cell clusters were dissociated to form single-cell suspensions. The cells (1×10^5^~1×10^6^) were suspended in 100 μL of PBS+0.02% EDTA and incubated with FVS780 for 15 min to label the dead cells. After washing and Fc-blocking, the cells were stained with the following three mAb panels for 30 min:

- Tumor cell panel: CD326 (EpCAM), CD45, CD90
- Lymphocyte panel: CD45, CD3, CD4, CD8, CD19, CD56
- Myeloid cell panel: CD11b, CD16, CD16, CD66b, CD163, CCR3(CD193).

After fixing and permeabilizing with Cytofix/Cytoperm, cells were stained with 0.1μg/mL DAPI and applied to BD LSR Fortessa™X-20. Antigen expression and ratios of well-characterized immune cell populations were analyzed using Flow Jo™ software (Becton–Dickinson). The tumor leukocyte ratio (TLR) was examined by calculating CD45(-)CD326(+) tumor cells/CD45(+)CD326(-) leukocytes as described previously.^17^

### Apoptosis Assay

Eosinophils were isolated from peritoneal fluids and circulating blood using a MACS separation kit (Miltenyi Biotec, Bergisch Gladbach, Germany). The eosinophil-mediated induction of apoptosis in tumor cells was assessed by flow cytometry, using a modified protocol as previously described.^18^ Briefly, OCUM-1 human GC cells (1 × 10^4^ cells) were seeded into 96-well U-bottom plates and co-cultured with purified eosinophils at various effector-to-target (E:T) ratios for 5 h at 37 °C. Subsequently, the cells were stained with FITC-Annexin-V and 7-AAD, along with PE-conjugated anti-CD45 mAbs and APC-conjugated anti-CD326 mAbs. The percentage of apoptotic OCUM-1 was measured by identifying annexin-V-positive cells within the gated CD45(-)CD326(+) population. Specific apoptosis was determined by subtracting the percentages of annexin-V(+) OCUM-1 in wells without eosinophils.

### Transcriptome Analysis

Eosinophils were isolated from the peritoneal fluid and peripheral blood of three patients who underwent one course of IP PTX chemotherapy, and total RNA was extracted from isolated eosinophils using the QIAamp RNA Blood Mini Kit from QIAGEN following the manufacturer’s protocol. RNA samples were diluted in 30 μL nuclease-free water. The RNA concentration was measured using a NanoDrop One spectrophotometer. RNA concentrations were confirmed to be >15 ng/μL for all samples. RNA sequencing was performed by Rhelixa, Inc. (Tokyo, Japan). Libraries were prepared using the SMART-seq messenger RNA HT Library Prep Kit (Takara Bio, Shiga, Japan) according to the manufacturer’s instructions and sequenced on an Illumina NovaSeq 6000 Sequencing System. For clustering, principal component analysis, differentially expressed genes analysis, gene ontology (GO) analysis, and Kyoto Encyclopedia of Genes and Genomes expression database pathway analysis were performed by Rhelixa, Inc. (Tokyo, Japan).

### Statistical Analysis

Statistical analyses were performed using GraphPad Prism version 8. Comparisons between groups were evaluated using the Mann–Whitney U test. Overall survival (OS) curves were estimated according to the Kaplan–Meier method and compared using the log rank test. For all analyses, the standard for a significant difference was set at *p* < 0.05.

## Results

### Proportions of Immune Cells in Peritoneal Cavity in Patients with PM from GC Before IP Chemotherapy

**Fig. 1:**
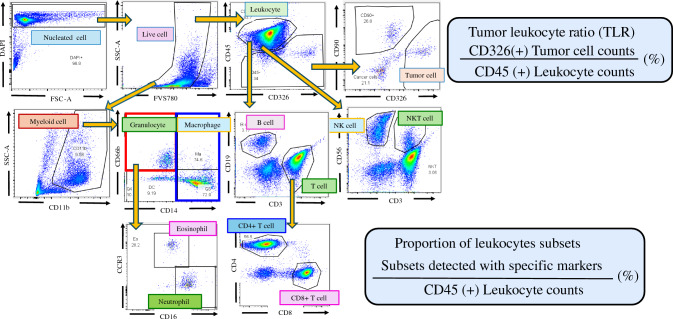
Gating strategy for lymphoid, myeloid, and tumor cells in peritoneal fluid. Among the DAPI(+) peritoneal free cells, dead cells were excluded by FVS780. Tumor cells and leukocytes were defined as CD45(–)CD326(+) and CD45(+)CD326(–), respectively, and the tumor-leukocyte ratio (TLR) was calculated. Within the leukocyte population, lymphoid and myeloid subsets were identified using standard surface markers

The lymphoid and myeloid cell phenotypes were determined using the gating strategy illustrated in Fig. 1, and their ratios to CD45(+) whole leukocytes were calculated. These data, obtained from 42 patients prior to IP chemotherapy, are summarized in Supplementary Fig. S1. The proportion of CD3(-)CD56(+) natural killer (NK) cells was lower (*p* = 0.002), whereas the proportion of CD14(+) macrophages was higher (*p* = 0.019) in female patients. The proportion of CD19(+) B cells was higher in patients with positive peritoneal cytology (CY(+)) than in those with negative cytology CY(-) (*P* = 0.026). In contrast, the proportions of CD3(-)CD16(+) NK cells (*P* = 0.077) and CD3(+)CD56(+) NKT cells (*p* = 0.034) tended to be lower in the CY(+) patients. However, the proportions of other lymphoid and myeloid cell subsets showed no significant correlation with macroscopic or histological tumor type, nodal status, Peritoneal Cancer Index score, or ascitic fluid volume.

### Change of the Proportions of Lymphocytes, Macrophages, and Their Subtypes After IP Chemotherapy

**Fig. 2:**
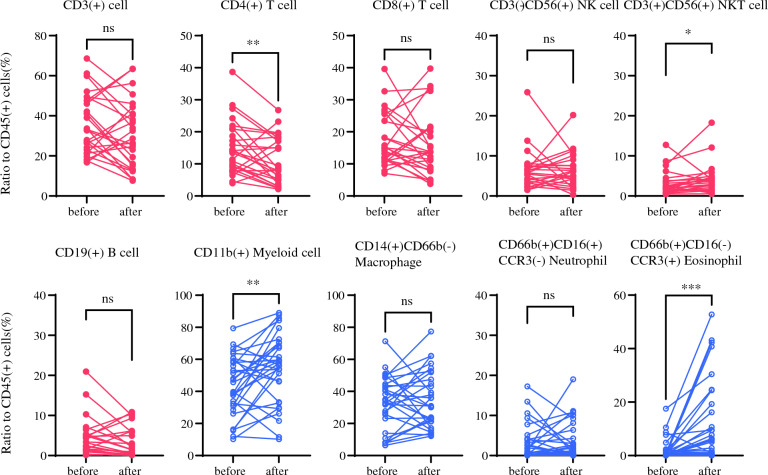
Changes in lymphoid and myeloid subsets before and after intraperitoneal (IP) chemotherapy in 31 patients. The proportions of CD4(+) and CD8(+) T cells, CD3(+)CD56(+) natural killer (NK) T-cell (NKT)-like cells, CD3(-)CD56(+) NK cells, and CD19(+) B cells, and CD11b(+) total myeloid cells, CD14(+)CD66b(-) macrophages, as well as CD66b(+)CD16(+)CCR3(-) neutrophils and CD66b(+)CD16(-) CCR3(+) eosinophils relative to CD45(+) were calculated in patients before and after 1–3 courses of combination chemotherapy. Statistical differences were evaluated using the Mann–Whitney U test. * p < 0.05, ** p < 0.01, *** p < 0.001

Peritoneal fluid samples were also collected from 31 patients after 1 to 3 cycles of combination chemotherapy, and the phenotypes of lymphoid and myeloid cells, relative to CD45(+) cells, were compared between pre- and post-chemotherapy samples (Fig. 2) Changes in the proportion of CD3(+) T cells varied among patients. Overall, a significant reduction in the proportion of CD4(+) T cells was observed in post-chemotherapy samples (median 8.6%, range 2.1–26.8) compared with pre-chemotherapy samples (median 13.7%, range 4.0–38.7, *p* = 0.0094). The proportion of CD19(+) B cells tended to be decreased (median 1.5%, range 0.02–10.9 compared with pre-chemotherapy levels, although this was not significant (median 3.23%, range 0.16–20.9, *p* = 0.095). Conversely, the proportion of CD3(+)CD56(+) NKT cells significantly increased in post-chemotherapy samples (median 3.6%, range 0.56–18.3) compared with pre-chemotherapy levels (median 2.0%, range 0.46–12.7, *p* = 0.014), despite the baseline proportions of B cells and NKT cells being lower than those of T cells.

In contrast, the proportion of CD11b(+) myeloid cells was significantly elevated following chemotherapy (median 56.3%, range 10.2–88.9) compared with baseline (median 45.7%, range 10.2–79.4, *p* = 0.0091) (Fig. 2). Among myeloid cells, no significant changes were observed in CD66b(-)CD14(+) macrophages or CD66b(+)CD16(+) CCR3(-) neutrophils. Notably, however, CD66b(+)CD16(-)CCR3(+) cells exhibited a marked increase in many patients. These cells exclusively expressed Siglec-8 and displayed high side scatter, indicative of eosinophils (Supplementary Fig. S2) Overall, the proportion of CD66b(+)CD16(-)CCR3(+) eosinophils significantly increased after chemotherapy compared with baseline (median 5.7%, range 0.01–52.8 vs. median 0.54%, range 0.01–17.5, *p* < 0.001) (Fig. 2).

### Eosinophil Increase Following One Course of IP Chemotherapy is Associated with Post-treatment Negative Cytology and Improved Patient outcomes

In 12 patients, eosinophil frequencies were evaluated at multiple time points during repeated IP chemotherapy. In most patients, eosinophil levels increased most prominently after the first treatment course, whereas this increase tended to be less clear after subsequent courses of IP chemotherapy (Supplementary Fig. S3). Therefore, we next investigated the association between eosinophil proportions following a single cycle of chemotherapy and clinical outcomes in a cohort of 27 patients for whom complete data were available. The patient demographics are shown in Supplementary Fig. S4.

**Fig. 3:**
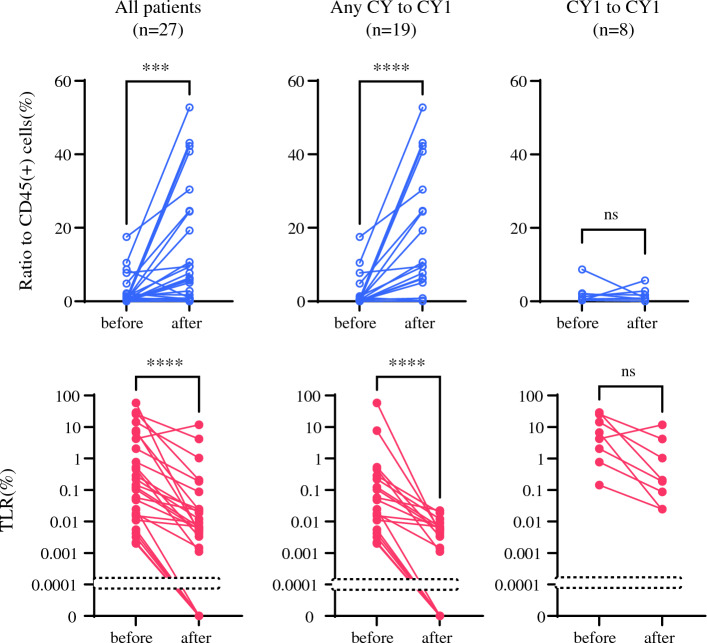
Eosinophil proportion (top) and tumor–leukocyte ratio (TLR) (bottom) before and after one intraperitoneal chemotherapy course in 27 patients. In total, 19 patients who were CY0 following treatment showed a significant increase in eosinophils with a marked reduction in TLR. In contrast, the eight patients who remained CY1 showed no detectable increase in eosinophils with less pronounced TLR. Statistical comparisons were performed using the Mann–Whitney U test. * p < 0.05, ** p < 0.01, *** p < 0.001

Among them, 19 patients achieved negative peritoneal cytology (CY0), whereas eight patients exhibited persistent positive cytology (CY1) after chemotherapy. The comparison of eosinophil proportions before and after treatment revealed notable differences between the two groups (Fig. 3). In patients who achieved CY0 after chemotherapy, the proportion of eosinophils remarkably increased (median 9.5%, range 0.01–52.8 vs. median 0.47%, range 0.015–17.53; *p* < 0.0001). Concomitantly, the TLR showed a marked reduction (median 0.0047%, range 0–0.23 vs. median 0.048%, range 0.0020–58.1; *p* < 0.0001), indicating a profound decrease in tumor cell burden within the peritoneal cavity. In contrast, for patients with persistent CY1, the decrease in TLR was less pronounced (median 0.20%, range 0.023–11.7 vs. median 5.5%, range 0.14–28.8; *p* = 0.078), and the proportion of eosinophils did not show a significant increase (median 0.91%, range 0.0035–5.7 vs. median 0.92%, range 0.26–8.7; *p* = 0.94). Supplementary Fig. S5 shows the changes in the CD11b(+) myeloid cell population analyzed using t-SNE in three representative patients with CY0 status post-treatment and one patient with persistent CY1 status.

**Fig. 4:**
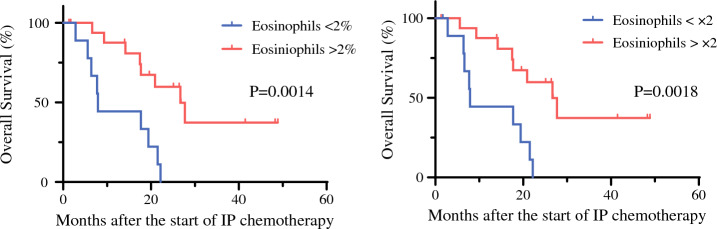
Overall survival by eosinophil proportion and increase after intraperitoneal (IP) chemotherapy (n = 27). Patients with **A** >2% eosinophils or **B** >2-fold increase showed better survival. Survival analysis was performed using the Kaplan–Meier method, and *p *values were determined using the log-rank test

The prognostic relevance of eosinophil dynamics was evaluated in this cohort. Pre-chemotherapy eosinophil proportions showed no correlation with OS. In contrast, post-chemotherapy eosinophil levels were strongly associated with survival outcomes. Using a threshold of 2%, patients with higher post-chemotherapy eosinophil proportions (n = 18) demonstrated significantly prolonged OS compared with those to lower proportions (n = 8; *p* = 0.0014) (Fig. 4, left). Moreover, patients who exhibited a >2-fold increase in eosinophil proportions after chemotherapy achieved markedly improved OS relative to those without such an increase (*p* = 0.0018) (Fig. 4, right).

Conversion surgery was performed in 16 of 18 patients (89%) with post-treatment eosinophil levels >2% after 3–6 courses of IP chemotherapy, compared with only one of nine patients (11%) in the counterpart group (*p* < 0.0001), suggesting a substantial impact on survival in this cohort. In a multivariate Cox regression analysis incorporating age, Peritoneal Cancer Index score, ascitic volume, and histological type of primary tumor biopsy before treatment, abdominal eosinophilia (>2%) emerged as the strongest prognostic factor for OS, although statistical independence could not be confirmed owing to the limited sample size of this cohort (Supplementary Fig. S6).

### Characteristics of Eosinophils Recovered from Peritoneal Fluids Following IP Chemotherapy

**Fig. 5:**
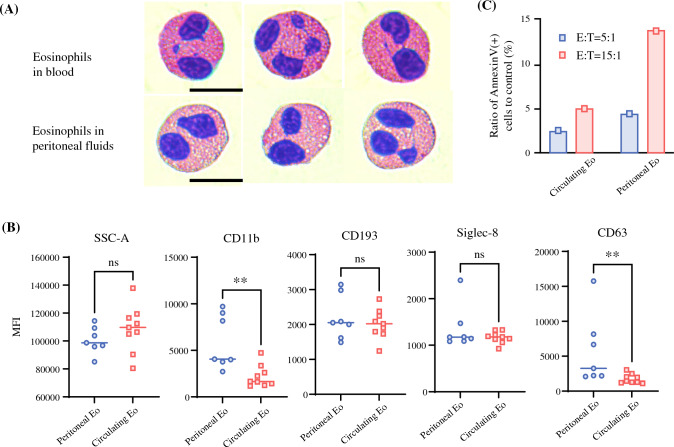
Characterization of peritoneal eosinophils after chemotherapy. **A** hematoxylin and eosin of peritoneal vs. blood eosinophils. **B** Flow cytometric comparison of CD11b, CD193, Siglec-8, CD63. Mean fluorescence intensities (MFI) for each antigen were compared between eosinophils obtained from peritoneal fluids and circulating blood. **C** Eosinophil-induced OCUM-1 apoptosis at indicated E:T ratios. Cells were stained with Annexin-V, 7-AAD, and monoclonal antibodies for CD45 and CD326, and analyzed via flow cytometry. Annexin-V(+) OCUM-1 cells were quantified within the gated CD45(-)CD326(+) population, with subtracting Annexin-V(+) percentages in eosinophil-free controls. Statistical differences were evaluated using the Mann–Whitney U test. ** p < 0.01

The eosinophils isolated from post-treatment peritoneal fluids using magnetic separation exhibited numerous large acidophilic granules within their cytoplasm, as observed using hematoxylin and eosin (H&E) staining. However, granule density appeared reduced compared with eosinophils derived from the peripheral blood of the same patients, with the presence of some vacuoles (Fig. 5A). Flow cytometric analysis showed that peritoneal eosinophils exhibited higher expression levels of CD11b and CD63 than their circulating counterparts. Additionally, the side scatter values of peritoneal eosinophils tended to be lower than those of circulating eosinophils. These findings suggested that peritoneal eosinophils exhibit a partially activated and degranulated phenotype (Fig. 5B). Furthermore, eosinophils isolated from peritoneal fluid following chemotherapy demonstrated a significantly greater capacity to induce apoptosis of OCUM-1, a human gastric cancer cell line, than eosinophils derived from peripheral blood (Fig. 5C).

### Transcriptomic Differences between Peritoneal and Circulating Eosinophils

**Fig. 6:**
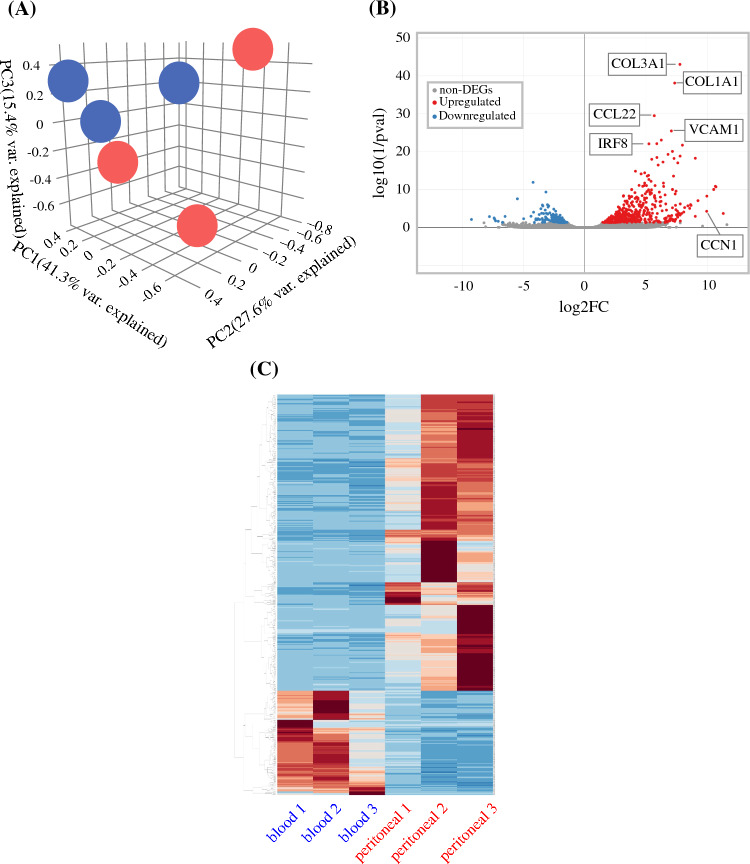
Transcriptomic comparison of peritoneal and circulating eosinophils (n = 3). **A** Principal component analysis of differentially expressed genes from RNA-seq. Each dot represents a single sample: peritoneal eosinophils (red) and circulating eosinophils (blue). **B** Volcano plot showing the distribution of genes differentially expressed between peritoneal and circulating eosinophils. Selected genes of interest are labeled. **C** Heatmap of differentially expressed genes in each group

Finally, we performed RNA sequencing to compare the gene expression profiles between peritoneal and circulating eosinophils. Principal component analysis demonstrated clustering within each group while clearly separating peritoneal from circulating eosinophils (Fig. 6A). Differential expression analysis revealed 565 transcripts with significant changes, including 418 genes upregulated and 147 downregulated in the peritoneal eosinophils (Fig. 6B). The heatmap of these transcripts showed highly consistent expression patterns within each group (Fig. 6C). GO enrichment analysis revealed that transcripts in peritoneal eosinophils were significantly enriched in biological processes related to cell adhesion (GO:0007155) and collagen fibril organization (GO:0030199), including genes such as *VCAM1*, *COL1A1*, and *COL3A1*. In addition, pathways associated with immune response (GO:0006955) and chemotaxis (GO:0006935) were enriched, represented by genes such as *CCL22*, *RARRES2*, *SLAMF7*, *LTBR*, *PRG4*, *CLEC10A*, *IRF8*, *CD1D*, *CD1C*, *XCR1*, *HLA-DRB1*, *HLA-DQA1*, and *CCN1* (Supplementary Figs. S7 and S8). Kyoto Encyclopedia of Genes and Genomes pathway analysis also showed that, in addition to genes related to complement and coagulation cascades, extracellular matrix–receptor interaction, and focal adhesion, genes associated with cytokine–cytokine receptor interaction were significantly upregulated in peritoneal eosinophils compared with circulating eosinophils (Supplementary Fig. S9).

## Discussion

In this study, we observed a marked increase in eosinophils within the peritoneal fluid of patients with GC with PM following combination chemotherapy, including IP PTX, particularly in those with favorable tumor responses. Eosinophil recruitment to tumors is known to be driven by type 2 helper cell-derived cytokines such as interleukin (IL)-4, IL-5, and eotaxins (CCL11, CCL24, and CCL26),^19–21^ as well as by damage-associated molecular patterns (DAMPs), including HMGB1,^22^ ATP,^23^ and IL-33.^18,24^ We examined whether the increase in eosinophil frequency was associated with clinical or experimental parameters prior to treatment; however, no significant correlations with any pretreatment factors were identified, and the precise mechanisms to induce abdominal eosinophilia remains unclear. Nevertheless, because PTX can induce immunogenic cell death through DAMP release,^25^ IP administration is likely to generate high local concentrations of DAMPs, thereby facilitating robust eosinophil recruitment.

Eosinophils have frequently been observed infiltrating tumor tissues in large numbers, a phenomenon known as tumor-associated tissue eosinophilia.^26,27^ In several malignancies, including GC, eosinophil accumulation is generally correlated with a favorable prognosis,^28–30^ although inverse associations have been reported in certain cancers,^31^^,^^32^ indicating that eosinophils can exert either anti- or pro-tumor effects depending on the microenvironment.^27,33^ In this study, post-treatment increases in peritoneal eosinophils were positively associated with cytological conversion to a negative status (CY0), conversion surgery, and improved survival, strongly supporting a functional antitumor role of eosinophils in PM.

The mechanisms underlying the anti-tumor effects of peritoneal eosinophils remain incompletely defined. Eosinophils can exert cytotoxic effects through the release of granular proteins, including major basic protein,^34^ eosinophilic cationic protein, eosinophil-derived neurotoxin,^35^ and granzyme B.^36^ More recently, IL-33–stimulated eosinophils have been shown to secrete extracellular vesicles that induce tumor cell-cycle arrest and mesenchymal–epithelial transition.^37^ In murine melanoma models, eosinophils contribute to tumor regression via direct cytotoxicity.^18–20,38^ In the present study, peritoneal eosinophils isolated following chemotherapy demonstrated reduced granule content, vacuolization, and increased expression of activation (CD11b) and degranulation (CD63) markers compared with circulating eosinophils. Transcriptomic analysis revealed the upregulation of genes associated with cell adhesion, extracellular matrix–receptor interaction, and focal adhesion. These processes are critical for stable target-cell engagement and polarization of granule proteins toward the immune synapse, thereby facilitating contact-dependent degranulation and tumor cell killing.^35,39^ Consistently, peritoneal eosinophils exhibited significant ability to induce apoptosis in GC cells. Collectively, these findings indicate that eosinophils are functionally activated and directly mediate the tumoricidal effects. Such activity may contribute to cytological conversion and improve outcomes in patients receiving IP chemotherapy.

However, recent studies have highlighted the multifaceted role of eosinophils in orchestrating antitumor immunity. Eosinophils have been shown to facilitate T-cell infiltration into tumor tissues and to enhance lymphocyte-mediated antitumor responses.^40,41^ Likewise, Lucarini et al.^18^ demonstrated that IL-33 suppresses tumor growth and metastasis through the coordinated accumulation of eosinophils and CD8⁺ T cells. In addition, eosinophils contribute to the recruitment of antitumor T cells and NK cells^42^ and dendritic cell maturation.^43^ In ovarian cancer, IP administration of IL-33 has been reported to delay the progression of PM through the cooperative activity of eosinophils, CD4⁺ T cells, B cells, and macrophages.^24^ Transcriptomic analyses in this study revealed that peritoneal eosinophils upregulated a broad array of genes linked to chemotaxis and antitumor immunity. This transcriptional profile suggests that peritoneal eosinophils may simultaneously amplify antitumor immune responses through recruitment and cross-talk with other immune subsets. Notably, our analyses were performed on eosinophils collected 3 weeks (one cycle) after the initiation of chemotherapy. Earlier timepoint sampling may yield further insights into the kinetics of eosinophil activation and its contribution to antitumor immunity.

In summary, we demonstrated that eosinophils with an activated phenotype accumulate in the peritoneal cavity after IP PTX, particularly in patients who have favorable responses. A previous case report described eosinophilic ascites after IP PTX in ovarian cancer with PM, which was associated with a favorable outcome,^44^ consistent with our findings. Systemic PTX administration is also known to frequently induce hypersensitivity reactions and peripheral eosinophilia.^45,46^ Furthermore, elevated peripheral eosinophil counts have been associated with improved survival in patients treated with immune checkpoint inhibitors.^47–49^ Collectively, these observations suggest that IP PTX may elicit an allergy-like immune response within the peritoneal cavity that contributes to antitumor activity against PM. Peritoneal eosinophilia may represent a promising biomarker for tumor response and patient outcome. Therapeutic strategies aimed at enhancing eosinophil recruitment and their antitumor activity may represent a novel approach to improving the efficacy of IP chemotherapy against peritoneal tumors.

## Supplementary Information

Below is the link to the electronic supplementary material.

Supplementary file1 (DOCX 37 KB)

Supplementary file2 (DOCX 1476 KB)

## Data Availability

The original contributions of this study are included in this article. Further inquiries can be directed to the corresponding author.
