# Supplementary material for: Intraperitoneal Paclitaxel-Induced Eosinophil Recruitment as a Potential Mediator of Tumor Response in Peritoneal Metastases from Gastric Cancer
Source: Ann Surg Oncol. 2026 Jan 29;33(6):5283–92. doi: 10.1245/s10434-025-19075-x (PMC13179214; doi:10.1245/s10434-025-19075-x)
Supplement: Supplementary file 1 — Supplementary file1 (DOCX 37 KB) [file 10434_2025_19075_MOESM1_ESM.docx]

Supplementary FigureS1. Supplementary Figure S1. Relation between the proportions of immune cells in peritoneal cavity and clinical and pathological characteristics in patients with PM from GC

PCI score:Peritoneal Cancer Index, Ascites was determined by CT image. Positive ascites cytology is defined as CY1, and negative as CY0. P classification : P1a: Peritoneal metastasis is limited to the stomach, greater omentum, lesser omentum, anterior lobe of the transverse colon mesentery, pancreatic capsule, and spleen, P1b: Metastasis is found in the peritoneum of the upper abdomen, P1c: Metastasis is found in the peritoneum of the middle and lower abdomen.tive."
