# Supplementary material for: Intraperitoneal Paclitaxel-Induced Eosinophil Recruitment as a Potential Mediator of Tumor Response in Peritoneal Metastases from Gastric Cancer
Source: Ann Surg Oncol. 2026 Jan 29;33(6):5283–92. doi: 10.1245/s10434-025-19075-x (PMC13179214; doi:10.1245/s10434-025-19075-x)
Supplement: Supplementary file 2 — Supplementary file2 (DOCX 1476 KB) [file 10434_2025_19075_MOESM2_ESM.docx]

**
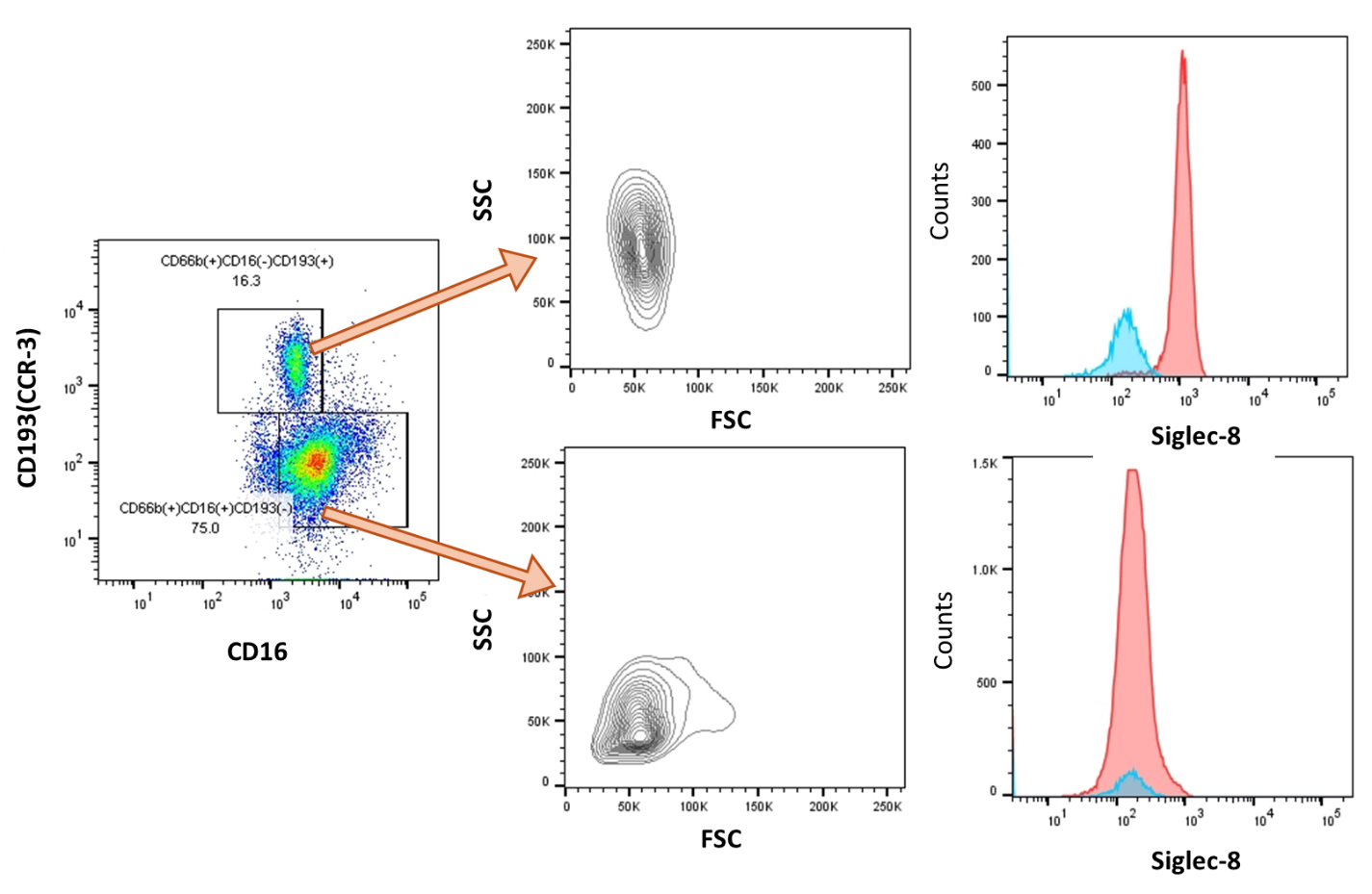
Supplementary Figure S2**

Flow cytometry forward scatter/side scatter (FSC/SCC) profiles and Siglec-8 expression patterns (Red line: Siglec-8; Blue line: isotype control) were analyzed in two distinct populations of CD66b(+)CD14(-) granulocytes isolated from peritoneal fluid in a representative patient. CD66b(+)CD16(-)CCR3(+) eosinophils exhibited exclusive Siglec-F positivity and demonstrated higher SCC values compared to CD66b(+)CD16(+)CCR3(-) neutrophils.

**Supplementary Figure S3**

**
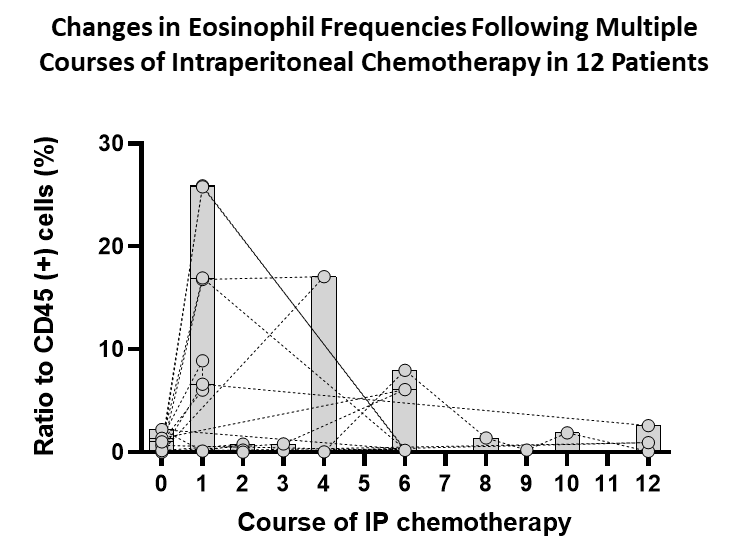
**

The proportions of CD66b(+)CD16(-) CCR3(+) eosinophils relative to CD45(+) were calculated at multiple time points during repeated IP chemotherapy in 12 patients and their temporal changes were shown.

**Supplementary Figure S5**


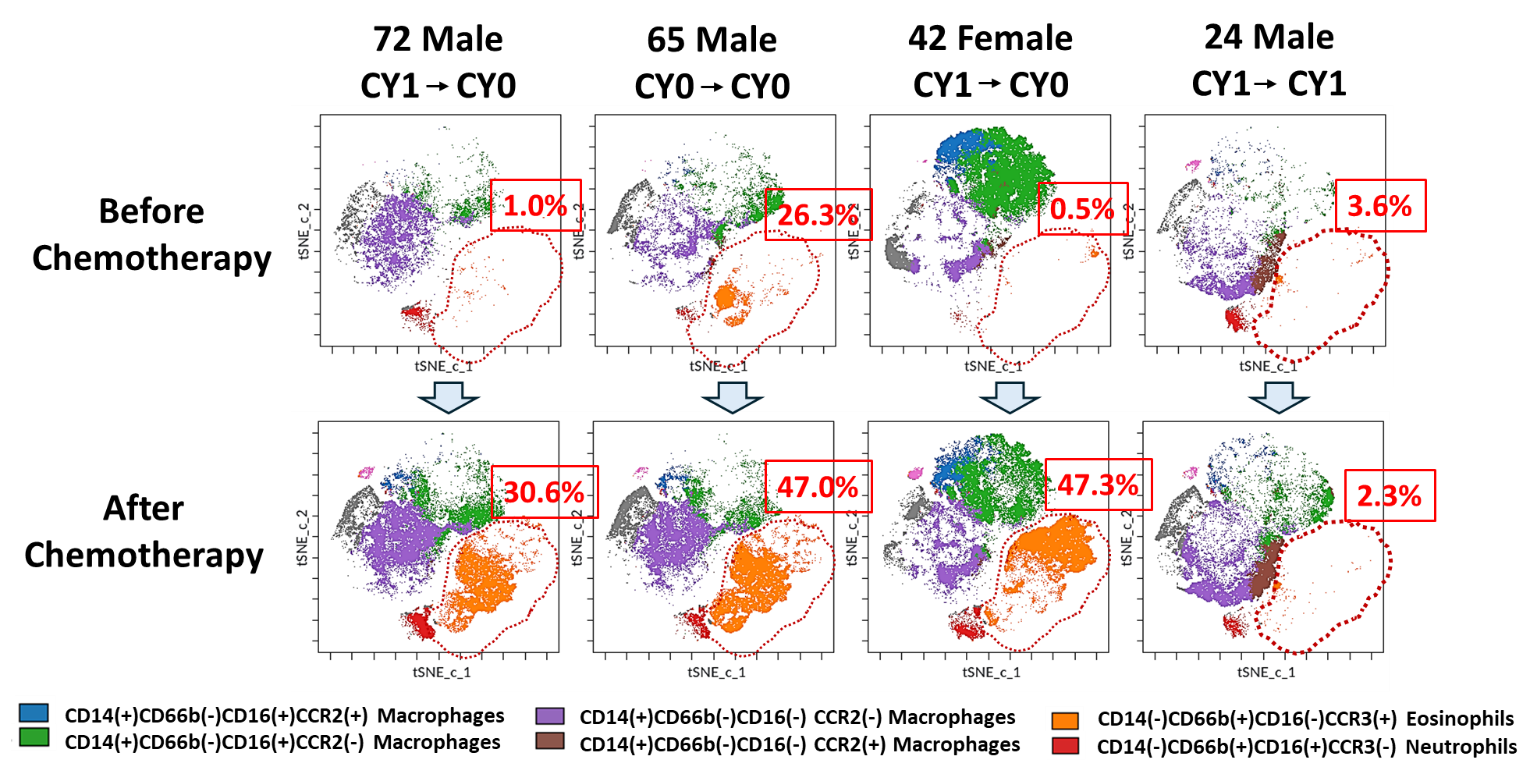


Marker expression was visualized using t-SNE mapping of CD11b(+) myeloid cells isolated from peritoneal fluid samples of four patients with peritoneal metastasis from gastric cancer, collected before and after one course of chemotherapy. Flow cytometry data from CD11b(+) myeloid cells of three patients with post-treatment CY0 status (left three panels) and one patient with persistent CY1 status (right panel) were exported, and t-SNE visualizations were generated using Cytobank software. Antigen expression levels for CD14, CD16, CD66b, and CCR3 were overlaid as color dimensions on the t-SNE maps. Each number represents the percentages to CD11b(+) myeloid cells.

**Supplementary Figure S6**

Hazard Ratio (HR) and p-values were calculated with Cox regression analysis.


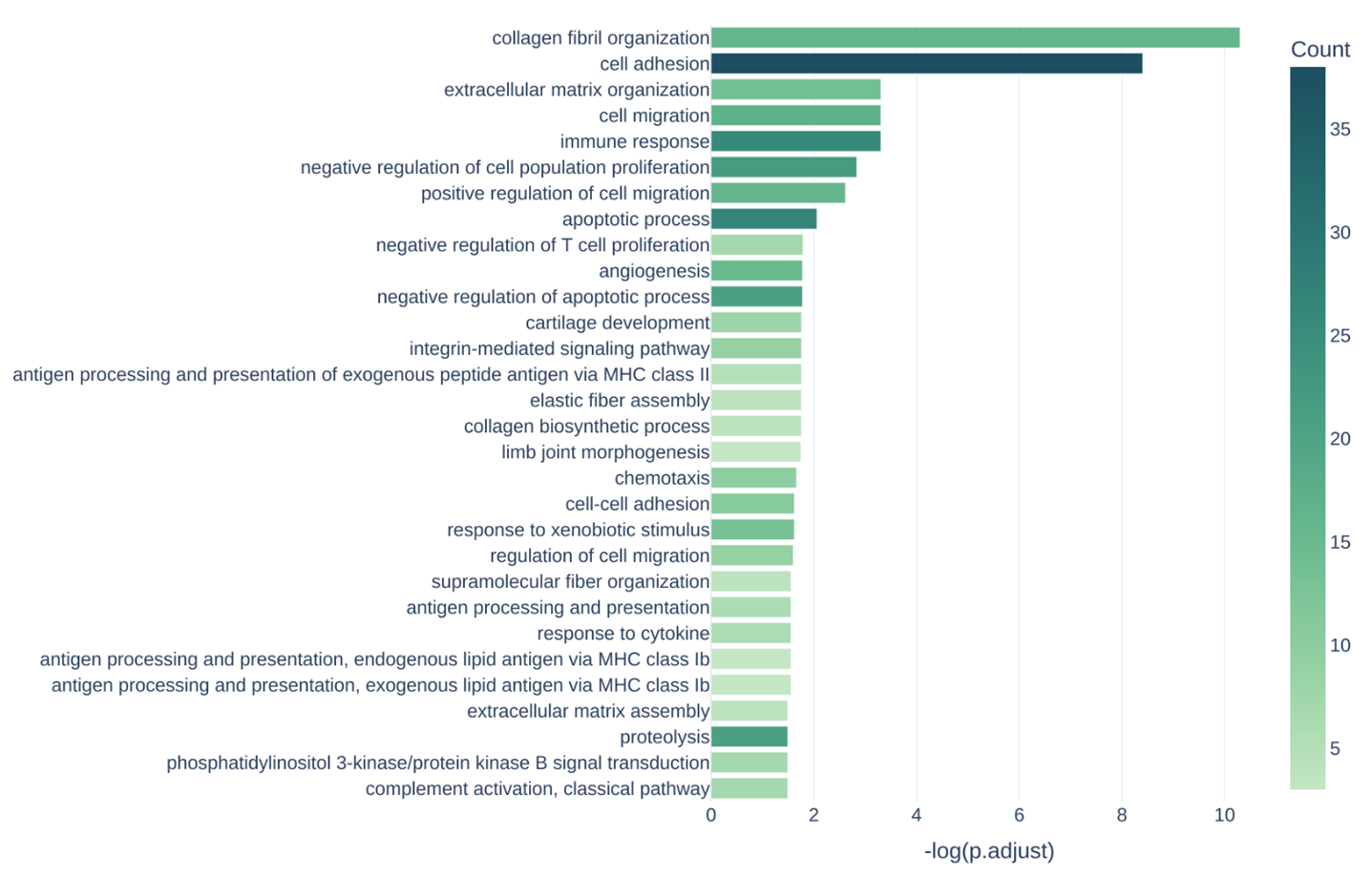
**Supplementary Figure S7**

Analysis of the biological process category of Gene Ontology pathways enriched in peritoneal eosinophils. Y-axis: GO terms. X-axis: negative logarithm of adjusted P-values from the GO enrichment analysis. Color: number of genes associated with each GO term. X-axis: negative logarithm of adjusted P-values from the enrichment analysis. Color: number of genes associated with each pathway.

**Supplementary Figure S8**

1. **(B) (C)**

**blood 1**

**blood 2**

**blood 3**

**Peritoneal 1**

**Peritoneal 2**

**l 2a**

**Peritoneal 3**

**blood 1**

**blood 2**

**blood 3**

**Peritoneal 1**

**Peritoneal 2**

**l 2a**

**Peritoneal 3**

**blood 1**

**blood 2**

**blood 3**

**Peritoneal 1**

**Peritoneal 2**

**l 2a**

**Peritoneal 3**

**
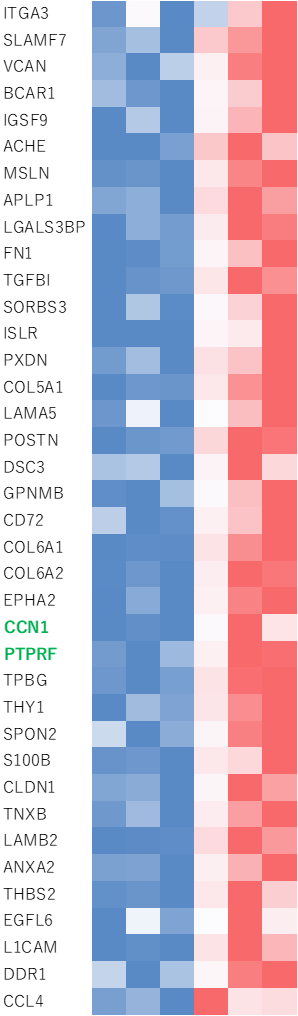
**
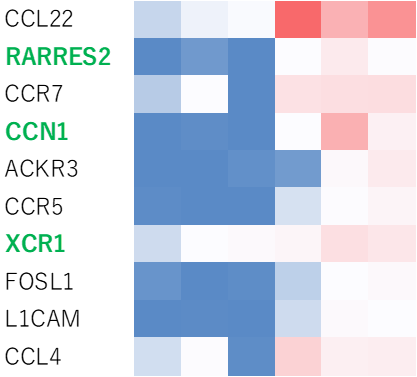

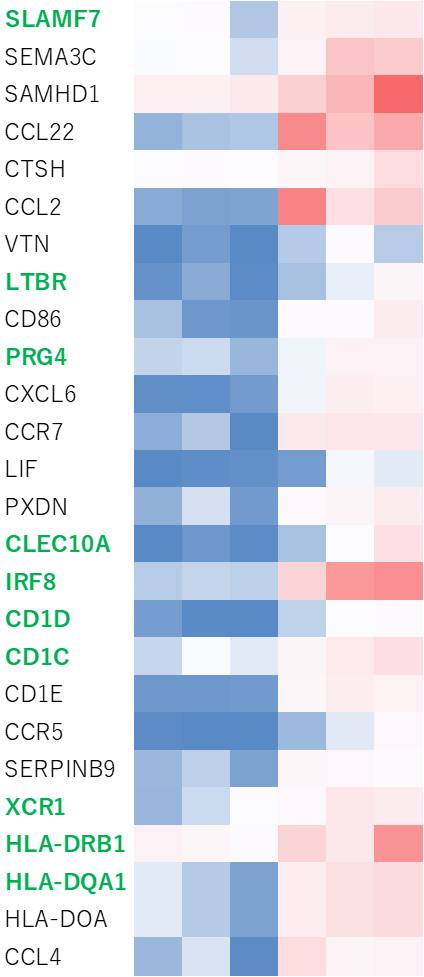


Heatmaps of selected differentially expressed genes associated with cell adhesion (A), immune response (B), and chemotaxis (C) in peritoneal eosinophils compared with circulating eosinophils. Red indicates upregulated genes, and blue indicates downregulated genes.

**
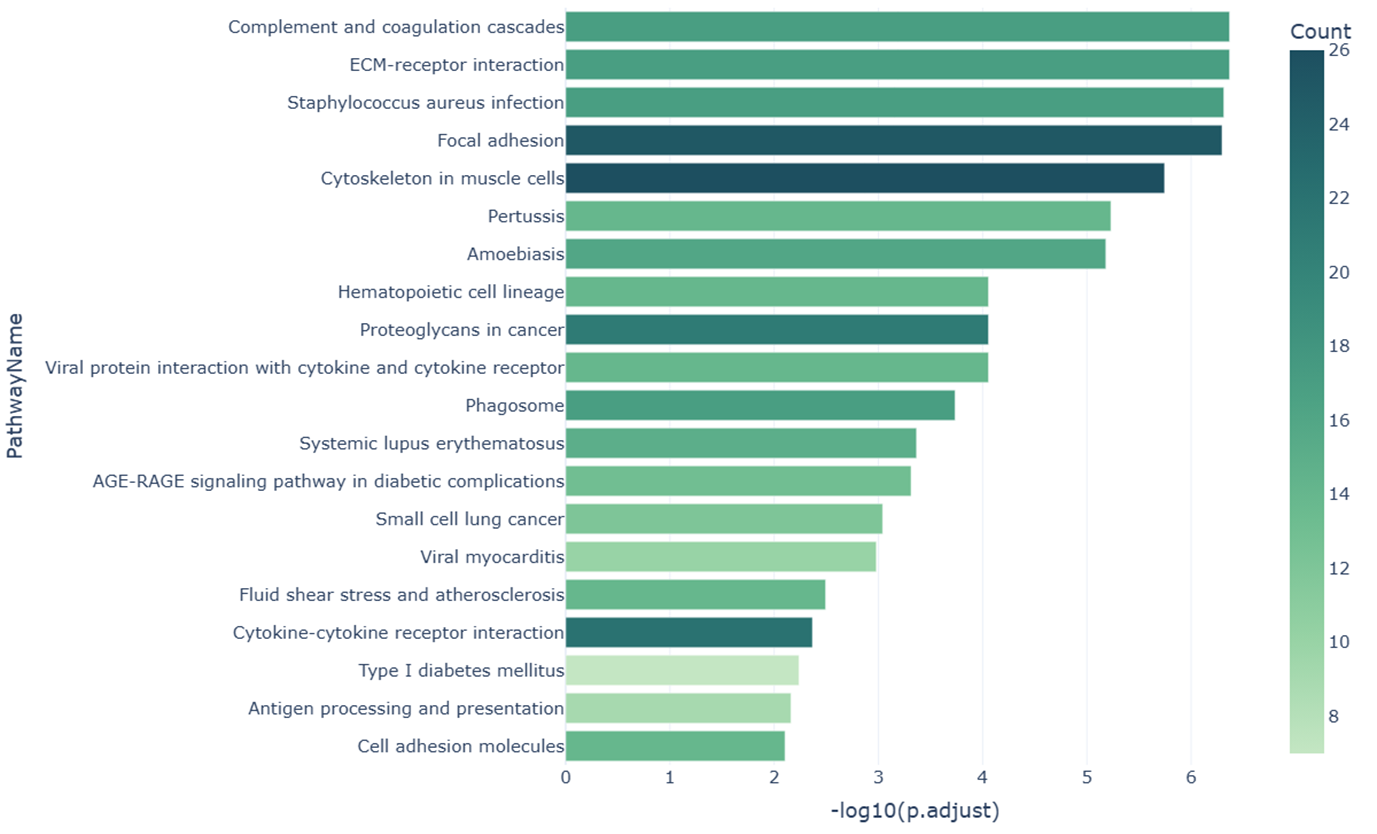
Supplementary Figure S9**

KEGG pathway analysis of pathways enriched in peritoneal eosinophils. Y-axis: pathway names. X-axis: negative logarithm of adjusted P-values from the enrichment analysis. Color: number of genes associated with each pathway.
